# Dopamine enhances willingness to exert effort for reward in Parkinson's disease

**DOI:** 10.1016/j.cortex.2015.04.003

**Published:** 2015-08

**Authors:** Trevor T.-J. Chong, Valerie Bonnelle, Sanjay Manohar, Kai-Riin Veromann, Kinan Muhammed, George K. Tofaris, Michele Hu, Masud Husain

**Affiliations:** a Department of Experimental Psychology, University of Oxford, Oxford, UK; b Nuffield Department of Clinical Neurosciences, John Radcliffe Hospital, Oxford, UK

**Keywords:** Dopamine, Effort, Reward, Decision-making, Parkinson's disease, PD, Parkinson's disease, MoCA, Montreal Cognitive Assessment, LARS, Lille Apathy Rating Scale, DASS, Depression Anxiety Stress Scale, UPDRS, Unified Parkinson's Disease Rating Scale, LE, Levodopa equivalence, MVC, Maximal Voluntary Contraction, ANOVA, Analysis of Variance

## Abstract

Parkinson's disease (PD) is traditionally conceptualised as a disorder of movement, but recent data suggest that motivational deficits may be more pervasive than previously thought. Here, we ask whether subclinical deficits in incentivised decision-making are present in PD and, if so, whether dopaminergic therapy ameliorates such deficits. We devised a novel paradigm in which participants decided whether they were willing to squeeze a hand-held dynamometer at varying levels of force for different magnitudes of reward. For each participant, we estimated the effort level at which the probability of accepting a reward was 50% – the effort ‘indifference point’. Patients with PD (*N* = 26) were tested ON and OFF their usual dopaminergic medication, and their performance compared to those of age-matched controls (*N* = 26). No participant was clinically apathetic as defined by the Lille Apathy Rating Scale (LARS). Our data show that, regardless of medication status, patients with PD chose to engage less effort than controls for the lowest reward. Overall, however, dopamine had a motivating effect on participants' choice behaviour – patients with PD chose to invest more effort for a given reward when they were in the ON relative to OFF dopamine state. Importantly, this effect could not be attributed to motor facilitation. We conclude that deficits in incentivised decision-making are present in PD even in the absence of a clinical syndrome of apathy when rewards are low, but that dopamine acts to eliminate motivational deficits by promoting the allocation of effort.

## 1 Introduction

Parkinson's disease (PD) is a prototypical model of striatal dysfunction. The accompanying dopaminergic depletion is traditionally considered one of the underlying mechanisms that contributes to the cardinal motor symptoms of bradykinesia, rigidity and tremor (Jankovic, 2008). Recently, however, some authors have proposed that at least some Parkinsonian motor symptoms may represent a deficit in ‘implicit’ motor motivation. For example, one study reported that patients with PD had similar kinematic parameters to controls, but were more likely to move slowly when the energetic demands of a movement increased (Mazzoni, Hristova, & Krakauer, 2007). They therefore conceptualised Parkinsonian bradykinesia as a shift in the balance between the perceived *reward* of reaching the target endpoint and the *amount of effort* required to achieve a movement of normal speed. Findings such as this suggest that motivational deficits may be more pervasive in PD than previously thought.

To determine if an action is worth initiating, one must evaluate the cost of that action – for example, the effort associated with it – against its potential rewards. Effort is generally considered aversive and, when given a choice, most animals will usually prefer actions that are less effortful (Salamone, Correa, Farrar, & Mingote, 2007; Walton, Kennerly, Bannerman, Phillips, & Rushworth, 2006). Thus, rewards which require less effort are generally preferred over rewards of identical value which are associated with greater effort (Hull, 1943). A number of animal studies have implicated dopamine in effort and reward valuation (Pasquereau & Turner, 2013). In rats, dopamine depletion decreases tolerance for effort, while drugs enhancing dopamine have the reverse effect (Salamone & Correa, 2002; Salamone et al., 2007). Human data regarding the involvement of dopamine on effort and reward integration remain relatively scarce, although there is a growing interest towards understanding the role of dopamine in cost-benefit integration (Frank, 2005; Wardle, Treadway, Mayo, Zald, & de Wit, 2011).

The pathognomonic striatal dysfunction in PD makes it an excellent model with which to study the effect of dopamine on incentivised decision-making in humans. It remains poorly understood how PD affects the valuation of an action's costs and benefits, and how that may subsequently affect choice behaviour. Although several studies in PD have examined impairments in decision-making and reward (e.g., Bódi et al., 2009; Cools, Barker, Sahakian, & Robbins, 2003; Czernecki et al., 2002; Frank, Seeberger, & O'Reilly, 2004; Mimura, Oeda, & Kawamura, 2006; Porat, Hassin-Baer, Cohen, Markus, & Tomer, 2014), relatively few have explicitly examined effort-based motivational deficits (e.g., Porat et al., 2014; Schmidt et al., 2008). Given the large animal literature postulating the role of striatal dopamine in incentivisation, we hypothesise that motivational deficits are likely present at least subclinically in PD, and independent of a clinical syndrome of apathy in which amotivation is a defining characteristic (Pluck & Brown, 2002). Moreover, we predict that dopamine should ameliorate these motivational deficits by promoting the allocation of effort.

Here, we report the results of a novel paradigm in which participants decided whether to accept or reject a potential reward based on the effort that would be required to obtain it. An important feature of our design was that it allowed us to focus on the effects of dopamine on participants' *choices*. This contrasts with many previous studies, especially those in animals, which have inferred the motivational effects of dopamine on behaviour by examining the effort manifest in the actions themselves (see Salamone et al., 2007 for review). By analysing participants' choices, we were able to calculate for each stake the effort level at which participants considered an action not worth pursuing – their ‘effort indifference points.’ We could then quantify the effect of PD and dopaminergic medication on shifting the position of these indifference points relative to healthy controls.

## 2 Material and methods

### 2.1 Participants

This study was approved by the local institutional review board, and patients did not receive financial compensation for their participation in the study. Patients with PD were recruited through a tertiary hospital and community support groups. All patients were reviewed by at least two consultant neurologists (TC and one other), and had a confirmed diagnosis of idiopathic PD. They were excluded if they had a history of stroke, depression, impulse control disorder, cognitive impairment [Montreal Cognitive Assessment (MoCA) score <26/30] or musculoskeletal disease that would have interfered with their ability to perform our task. Patients were on levodopa-containing compounds (*n* = 10), dopamine agonists (*n* = 5, including pramipexole, ropinirole, rotigotine), or combinations of both (*n* = 11). Clinical severity was assessed with the Unified Parkinson's Disease Rating Scale (UPDRS) (Fahn et al., 1987). We screened for apathy and depression with the Lille Apathy Rating Scale (LARS) (Sockeel et al., 2006) and Depression Anxiety Stress Scale (DASS) (Brown, Korotitsch, Chorpita, & Barlow, 1997; Lovibond & Lovibond, 1995), respectively. Table 1 summarises the characteristics of our final sample of 26 patients. An equal number of age- and education-matched controls was recruited through the local participant pool. Control participants were excluded if they had a history of neurological illness, but exclusion criteria were otherwise identical to those for patients.

### 2.2 Method

Participants were seated in front of a computer running Psychtoolbox (http://psychtoolbox.org) implemented in Matlab (MathWorks, USA). They registered their responses using two hand-held dynamometers (SS25LA, BIOPAC Systems, USA).

At the beginning of each session, the dynamometers were calibrated to each participant's maximal voluntary contraction (MVC). Participants alternately squeezed the left and right dynamometers as strongly as possible, and the maximum contraction reached over three trials was taken as each participant's MVC for that hand. This procedure normalised subsequent responses to each participant's maximum force.

**Fig. 1:**
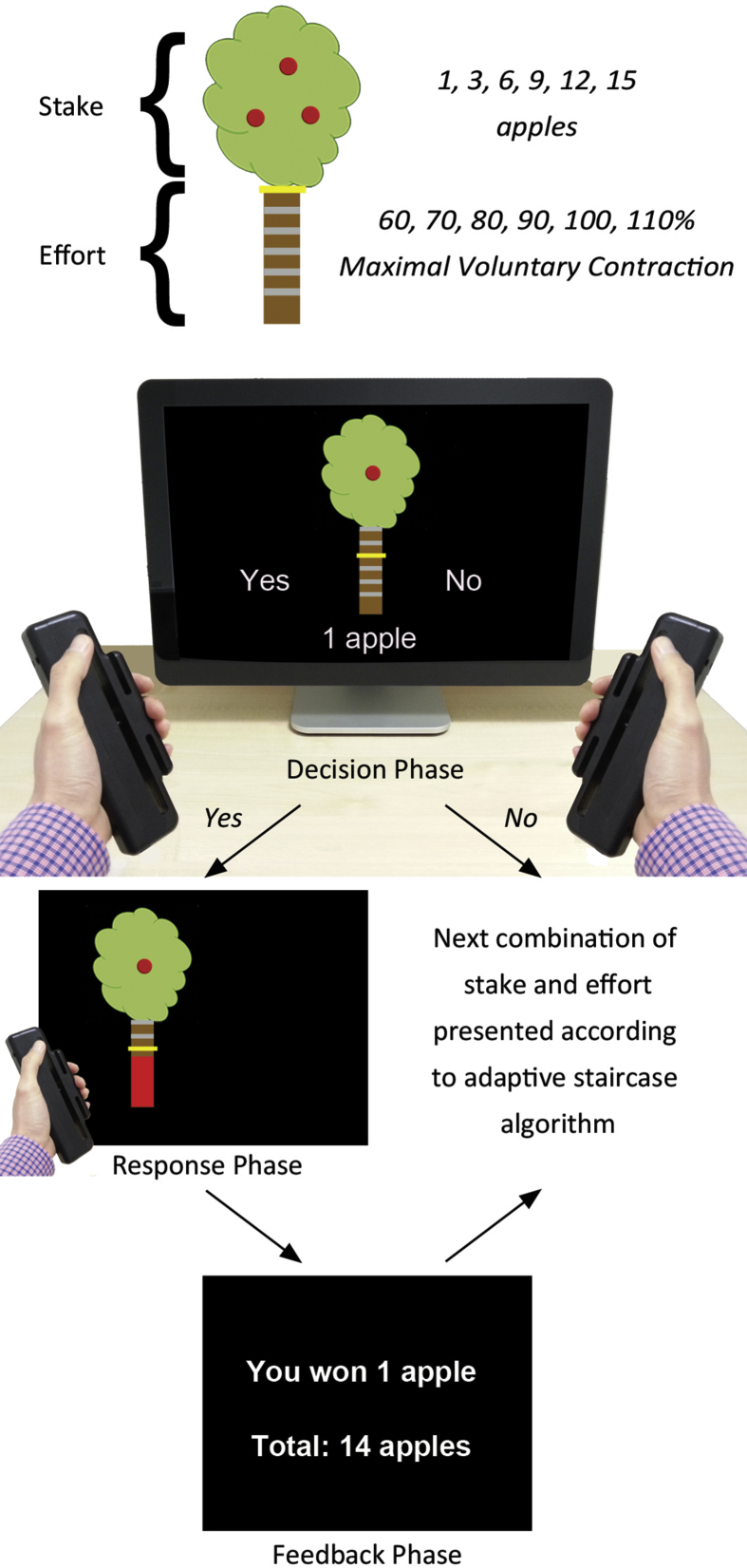
**Summary of a typical trial**. Stakes were indicated by the number of apples on the tree (1, 3, 6, 9, 12, 15), while the associated effort was indicated by the height of a yellow bar positioned at one of six levels on the tree trunk (corresponding to MVCs of 60%, 70%, 80%, 90%, 100%, 110%). On each trial, participants decided whether they were willing to exert the specified level of effort for the specified stake. If they judged the particular combination of stake and effort to be ‘not worth it,’ they selected the ‘No’ response. If, however, they decided to engage in that trial, they selected the ‘Yes’ response, and then had to squeeze a hand-held dynamometer with a force sufficient to reach the target effort level. Participants received visual feedback of their performance, as indicated by the height of a red force feedback bar. To reduce the effect of fatigue, participants were only required to squeeze the dynamometers on 50% of accepted trials. At the conclusion of each trial, participants were provided with feedback on the number of apples gathered.

During the experiment, participants were presented with cartoons of apple trees, and were instructed to accumulate as many apples as possible based on the combinations of stake and effort that were presented (Fig. 1). Potential rewards were indicated by the number of apples on the tree (1, 3, 6, 9, 12, 15), while the associated effort was indicated by the height of a yellow bar positioned on the tree trunk, and ranged over six levels as a function of participants' MVCs (60%, 70%, 80%, 90%, 100%, 110%). By referencing the effort levels in each session to each individual's maximum force, we were able to normalise the difficulty of each level across sessions and across individuals. Participants were familiarised with the effort required for each level prior to commencing the experiment.

On each trial, participants had to decide whether they were willing to exert the specified level of effort for the specified stake. If they judged the particular combination of stake and effort to be ‘not worth it,’ they selected the ‘No’ response, and the next trial would commence. If, however, they decided to engage in that trial, they selected the ‘Yes’ option. The tree would subsequently reappear on the left or right of the screen (selected at random), corresponding to the hand to be used for response execution. Participants then had five seconds to squeeze the dynamometer to reach the target effort level. Apples could only be acquired if the target effort level was reached; if participants failed to do so, no apples were received. If they rejected a particular combination of effort and reward, they were instructed that a different tree would subsequently appear and they were to proceed with the same process. At the conclusion of the trial, they received feedback on their performance. Combinations of stake and effort were presented according to an adaptive staircase algorithm (see Supplementary Material).

After an initial practice block of 36 trials, participants completed five experimental blocks of 36 trials, separated by rest breaks. They were tested in two sessions approximately one week apart. In one (‘ON’) session, patients were tested while taking their usual dopaminergic medication; and, in the other (‘OFF’), patients were tested after overnight withdrawal of medication. The order of ON and OFF sessions was counterbalanced across patients. Control participants performed two identical sessions to exclude the possibility of strategic changes across sessions.

## 3 Results

**Fig. 2:**
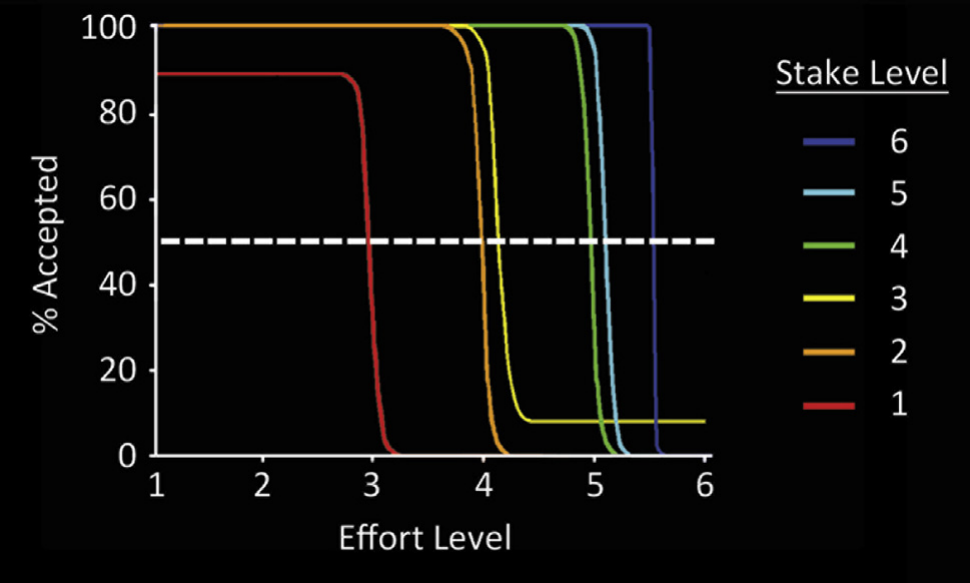
**An example of the fitted probability functions for a representative participant**. Logistic functions were used to plot the probability of engaging in a trial as a function of the effort level for each of the six stakes. Each participant's effort indifference points – the effort level at which the probability of engaging in a trial for a given stake is 50% (indicated by the dashed line) – were then computed.

For each stake, we estimated the effort level at which the probability of accepting an offer was 50% (i.e., the effort ‘indifference point’). For each participant, we fitted a logistic function to the choice probability data at each effort level (Fig. 2). The effort indifference points thus derived for each participant were then plotted against their corresponding stake magnitudes. We then compared the effort indifference points for PD ON, PD OFF and controls with repeated-measures ANOVAs.

### 3.1 Control data

**Fig. 3:**
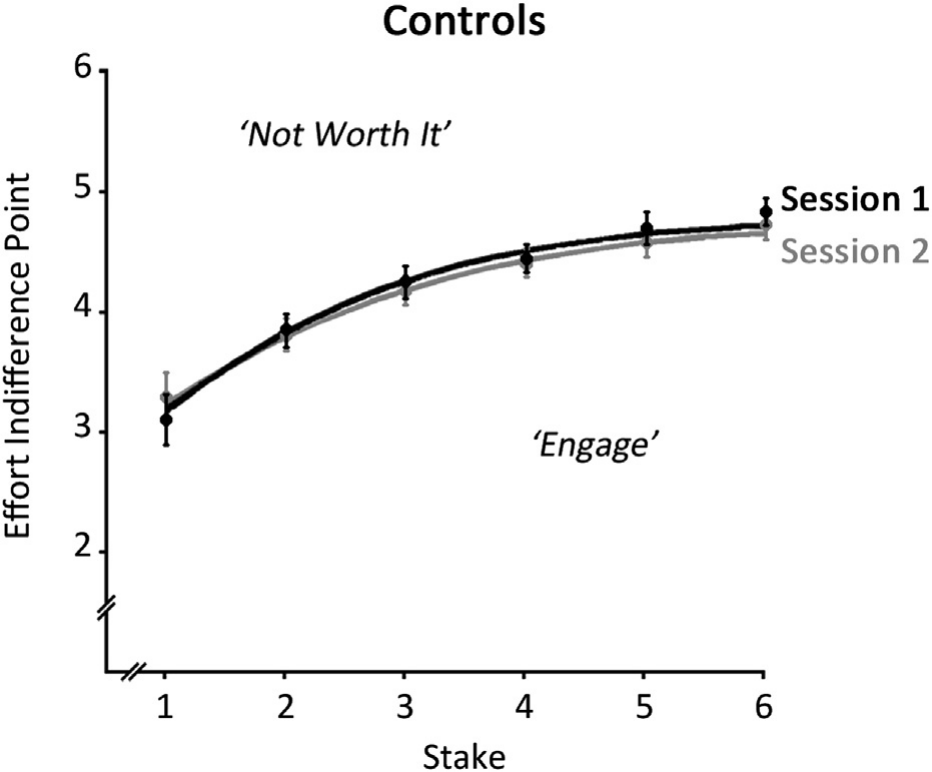
**Effort indifference points plotted as a function of stake for healthy controls in Sessions 1 and 2**. Effort indifference points divide the stake-effort space into a sector in which participants are willing to engage in an effortful response (below the curve) from a sector that is judged ‘not worth the effort’ (above the curve). Control performance was identical between sessions 1 and 2. Error bars indicate ±1 SEM.

First, we ensured that control performance did not differ across testing sessions (Fig. 3). A repeated-measures ANOVA on effort indifference points with the factors of Session (First, Second) and Stake (Levels 1–6) showed a significant main effect of Stake [*F*(5, 125) = 47.90, *p* < .001], with Bonferroni-corrected contrasts revealing significant differences at each successive Stake Level (all *p* < .05). Importantly, neither the main effect of Session [*F*(1, 25) = .59] nor its interaction with Stake [*F*(5, 125) = 1.54] was significant, indicating no differences in control performance across Sessions 1 and 2. We therefore collapsed the control data across the two sessions for subsequent analyses.

### 3.2 Patient data – ON versus OFF

**Fig. 4:**
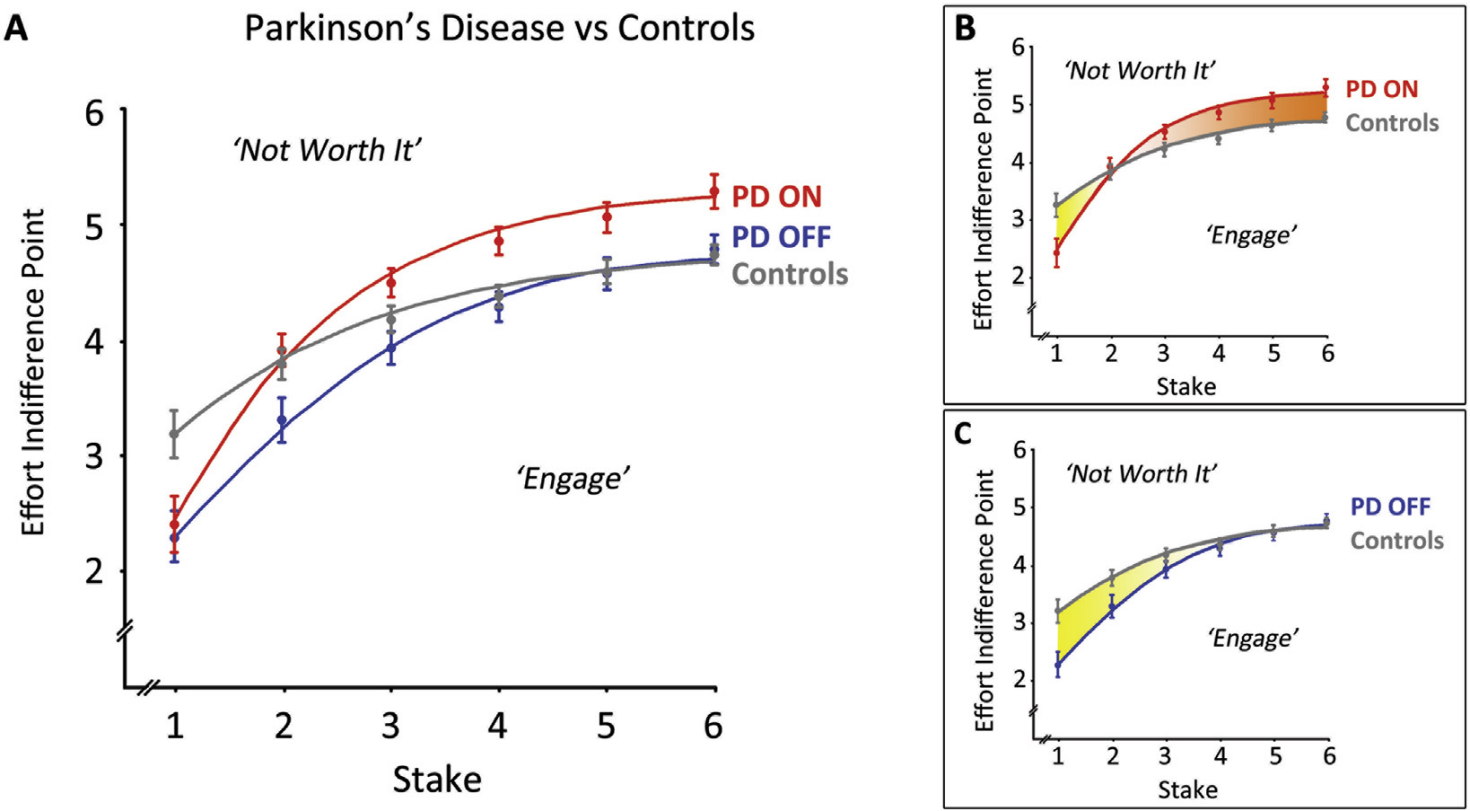
**Effort indifference points plotted as a function of stake for patients and controls. (A)** Regardless of medication status, patients had significantly lower effort indifference points than controls for the lowest reward. However, for high rewards, effort indifference points were significantly higher for patients when they were ON medication, relative not only to when they were OFF medication, but even compared to healthy controls. **Inset**: For clarity, PD data are replotted against control performance for patients **(B)** ON medication and **(C)** OFF medication. Shading denotes effort indifference points being greater for patients than controls (orange), or less for patients than controls (yellow). Error bars indicate ±1 SEM.

To compare the effect of drug on effort indifference points, we performed a similar two-way repeated-measures ANOVA, with the factors of Drug (ON, OFF) and Stake (1–6) (Fig. 4A). This revealed a significant main effect of Drug, *F*(1, 25) = 25.9, *p* < .001, such that patients ON medication were willing to invest more effort than those OFF, as reflected by a higher mean effort indifference point (M 4.34 ± SE .10 *vs* 3.89 ± .13). The main effect of Stake was also significant, *F*(5, 125) = 111.2, *p* < .001, with Bonferroni-corrected contrasts demonstrating significant differences between all pairings of Stake (*p* < .001). The interaction between Drug and Stake was not significant [*F*(5, 125) = 1.26].

To determine if maximal force output was modulated by dopamine, we compared MVCs ON and OFF medication. Importantly, they were not significantly different [OFF 354 ± 24*N vs* ON 360 ± 23*N*, *t*(25) = −1.34]. There was also no significant effect of time-on-task, which we used to examine the effect of fatigue on motor performance (see Supplementary Material). Furthermore, there was no correlation between shifts in effort indifference points and improvements in motor severity on the motor subscale (Part III) of the UPDRS (*r* = .22, *p* = .28; see Supplementary Material). Thus, the shift of effort indifference points ON medication was not simply attributable to a capacity to exert greater force or reductions in motor severity.

Given the association between dopamine and impulse control disorders (Weintraub et al., 2010), could the incentivising effect of dopamine be mediated by lower risk aversion? We analysed the proportion of trials in which patients engaged in effort levels beyond their capacity to perform (i.e., Effort Level 6, or 110% MVC). Importantly, there was no significant difference in this parameter ON versus OFF medication [*t*(25) = −1.59]. Furthermore, there was no effect of drug on the proportion of accepted trials in which patients failed to reach the target effort level [*t*(25) = .17], and no effect of drug on failure rates or trial history (see Supplementary Material).

### 3.3 Patient versus control data

Next, we compared patient performance ON medication with that of controls (Fig. 4B). An ANOVA showed a significant effect of Stake [*F*(5, 250) = 106.96, *p* < .001] but not of Group, which was qualified by a significant interaction [*F*(5, 250) = 9.62, *p* < .001]. Patients ON dopamine invested less effort than controls for the lowest Stake (2.42 ± .24 *vs* 3.19 ± .19, *p* < .05). However, quite the opposite was found for higher Stakes (levels 4–6), at which controls were actually willing to exert less effort than patients ON medication (Stake Level 4, ON 4.89 ± .11 *vs* Control 4.40 ± .11, *p* < .005; Level 5, ON 5.05 ± .12 *vs* Control 4.62 ± .12, *p* < .05; Level 6, ON 5.26 ± .13 *vs* Control 4.75 ± .13, *p* < .01). Notably, there was no significant difference in MVCs between patients ON medication and controls [Patients 360 ± 23*N vs* Controls 350 ± 24*N*, *t*(50) = .31].

For patient performance OFF medication versus controls (Fig. 4C), the analogous ANOVA demonstrated a significant effect of Stake [*F*(5, 250) = 111.90, *p* < .001], with a non-significant main effect of Group [*F*(1, 50) = 2.70]. Again, the two-way interaction was significant [*F*(5, 250) = 6.12, *p* < .001], with Bonferroni-corrected comparisons revealing that patients OFF medication were willing to expend less effort than controls, but only for the *lowest* two stakes (Stake Level 1, OFF 2.31 ± .21 *vs* Control 3.19 ± .19, *p* < .005; Level 2, 3.33 ± .16 *vs* 3.80 ± .16, *p* < .05). MVCs between patients OFF medication and controls were not significantly different [Patients 354 ± 23*N vs* Controls 350 ± 24*N*, *t*(50) = .129].

## 4 Discussion

Few studies to date have examined impairments in effort-based decision-making in PD (e.g., Porat et al., 2014; Schmidt et al., 2008). Our data reveal two key findings. First, patients with PD, regardless of medication status, were willing to invest less effort than their healthy counterparts for the lowest reward. Second, dopamine exerted a motivating influence on choice behaviour. Specifically, patients with PD chose to invest more effort for a given stake when they were ON medication relative to OFF. Importantly, the incentivising effect of dopamine cannot simply be due to motor facilitation, as there were no significant differences in MVC across drug session, or between patients and controls. Furthermore, the shift in effort indifference points from OFF to ON was not correlated with improvements in clinical motor severity as measured by the motor section of the UPDRS.

A notable feature of our paradigm, and one of its significant strengths, is that it allowed us to dissect out choice behaviour from motor preparation and execution. Many studies, in particular those in animals, infer the effect of dopamine on effort by observing the effort manifest in the behaviour itself (see Salamone et al., 2007 for review). A recent study in healthy adults, for example, reported that dopamine augments response vigour in proportion to average reward rate (Beierholm et al., 2013). In contrast to these previous studies, however, our paradigm demonstrates that the incentivising effect of dopamine is evident even during *choice behaviour* – i.e., *prior to* an action being initiated.

The question of how dopamine modulates aberrant cost-benefit integration in PD has not been extensively explored. The finding that patients ON medication were willing to exert greater force relative to OFF supports animal data showing that increasing dopaminergic tone enables high-effort behaviours and increases tolerance of effort expenditure (Cagniard et al., 2006; Niv, Daw, Joel, & Dayan, 2007; Robbins & Everitt, 1992; Wardle et al., 2011). Critically, this incentivising effect of dopamine is independent of any motor changes which might have occurred between the OFF and ON sessions. This is an important consideration, given that a recent study in PD found that the greater number of key-presses that patients exerted for reward when medicated was related to an improvement in their motor symptoms (Porat et al., 2014). Our study builds on these previous findings by showing that the motivational effect of dopamine on effort-based choices can occur *independent* of motor facilitation, as measured by either motor strength (MVC) or the clinical severity of motor signs (UPDRS).

Studies of disordered motivation in PD often focus on clinically apathetic patients (e.g., Dujardin et al., 2007). Here, we show that patients with PD, who were neither clinically apathetic nor depressed, and regardless of medication status, were less motivated than controls to invest effort when the rewards were low. This confirms that Parkinsonian striatal dysfunction is sufficient to cause an imbalance in the estimation of an action's expected value, and is consistent with animal studies showing that dopamine antagonism or depletion reduces willingness to work for reward (Salamone et al., 2007). Although we only found a reduction in motivation for the lowest levels of reward, any potential differences at higher stakes in the comparison of PD OFF versus controls could very well have been obscured by a saturation effect at the highest levels of effort. It should also be noted that our finding of lower effort indifference points in patients versus controls for low stakes occurred despite the LARS scores between the two groups being statistically similar and within the normal range. This result therefore emphasises that motivational deficits may be present subclinically in PD for low rewards, but that they are detectable with a sufficiently sensitive measure.

Finally, it is worth considering why participants in our task may have been willing to trade effort for fictive rewards. There is of course a considerable literature that supports the view that effort carries a value cost, and discounts the subjective value of potential rewards (e.g., Botvinick, Huffstetler, & McGuire, 2009). Complementing this literature is a considerable volume of evidence showing that real and fictive rewards are discounted similarly in behavioural paradigms (Hinvest & Anderson, 2010; Madden, Begotka, Raiff, & Kastern, 2003; Matusiewicz, Carter, Landes, & Yi, 2013). Furthermore, fMRI studies have shown that real and fictive rewards recruit overlapping neural regions (Bickel, Pitcock, Yi, & Angtuaco, 2009). In light of these findings, we therefore expected our participants to discount effort even in the presence of fictive rewards, as they in fact ultimately did.

Together, our findings show that deficits in incentivised decision-making are present in PD when rewards are low even in the absence of a clinical syndrome of apathy, but that dopamine acts to ameliorate motivational deficits by promoting the allocation of effort. This echoes recent reports that Parkinsonian movement shares many attributes with healthy behaviour (Desmurget et al., 2004), with a reduced motor drive being central to certain Parkinsonian motor symptoms (Kojovic et al., 2014; Mazzoni et al., 2007). The pervasiveness of motivational impairments in PD invites reconsideration of the degree to which Parkinsonian hypokinesia is due simply to motor dysfunction versus a primary motivational deficit. These contributions are not mutually exclusive, and both might be important in determining the surface manifestations of dopaminergic deficits in PD.

## Figures and Tables

**Table 1:** Summary of participant demographics (means ± SD).

	Patients with PD	Healthy controls	Group difference
*N*	26	26	–
Age (years)	66.6 (±6.8)	66.2 (±6.4)	*t*(50) = .23, *p* = .82
Gender (M:F)	17:9	15:11	*χ*^2^ = .08, *p* = .78
LARS^a^	−28 (±4.2)	−29 (±5.7)	*U* = 277, *p* = .23
Depression Score on DASS^b^	2.00 (±2.23)	1.5 (±1.84)	*U* = 295, *p* = .41
MoCA Scores^c^	28.2 (±1.3)	28.2 (±1.7)	*t*(50) = .09, *p* = .93
UPDRS III (ON, OFF)^d^	ON: 21.6 (±11.7) OFF: 31.9 (±13.6)	N/A	–
Hoehn & Yahr Stage^d^	1.85 (±.54)	N/A	–
Disease duration (years)	5.1 (±3.1)	N/A	–
Levodopa equivalence (mg)^e^	538 (±275)	N/A	–
Interval between sessions (days)	7.8 (±1.7)	7.2 (±.8)	*t*(50) = 1.51, *p* = .14
Average time since last dose (hours)	ON: 2.28 (±.97) OFF: 13.4 (±3.4)	N/A	–

a Normal range < −16 (Sockeel et al., 2006).

b Normal range = 0–9 (Lovibond & Lovibond, 1995).

c MoCA normal range 26–30.

d Clinical severity was assessed with the motor section (Part III, items 18–31) of the Unified Parkinson's Disease Rating Scale (UPDRS) (Fahn et al., 1987) and the modified Hoehn and Yahr scale. See Supplementary Table 1 for a full summary of patients' UPDRS data.

e Levodopa equivalence (LE) scores were calculated based on standard formulae (Tomlinson et al., 2010). Patients were on levodopa-containing compounds (*n* = 10), dopamine agonists (*n* = 5), or combinations of both (*n* = 11).
