# Supplementary material for: Dopamine enhances willingness to exert effort for reward in Parkinson's disease
Source: Cortex. 2015 Aug;69:40–6. doi: 10.1016/j.cortex.2015.04.003 (PMC4533227; doi:10.1016/j.cortex.2015.04.003)
Supplement: Supplementary file 1 [file mmc1.doc]

**Supplementary Material**

**S1. Supplementary Method**

For the main experiment, participants were instructed that the aim of this task was to gather as many apples as they could based on the combinations of effort and reward presented on each trial. Prior to commencing the task, participants were first familiarised with the amount of force required to achieve each effort level. During these preliminary trials, images of trees without apples were presented. They were told that the height of the horizontal bar on the tree trunk was proportional to the amount of force they would need to exert in the main experiment. However, they were not explicitly informed about the percentage MVC corresponding to each level. In this preliminary phase, participants had the opportunity to familiarise themselves with the amount of force required for each effort level by squeezing the dynamometers on separate trials to attempt to achieve each target effort level (two familiarisation trials per effort level).

Trials were presented according to an **adaptive staircase algorithm**, in which combinations of stake and effort were presented depending on participants’ previous choices . If a particular combination of stake and effort was declined on one trial, a higher stake or lower effort level was presented on a subsequent trial (stake and effort levels were adjusted alternately). The opposite would occur if a combination was accepted. Three randomly interleaved staircases were used so that participants were unaware of the algorithm.

The advantage of such a design, in contrast to the approach of randomly sampling the entire stake-effort space, is that it substantially reduced any effect of learning on task performance – an important consideration given that dopamine is thought to be involved in reinforcement and associative learning . By using a staircase algorithm, we were able to converge efficiently on participants’ indifference points, which were therefore derived independently of any associative learning between stimulus and reward, or force and effort.

In estimating participants’ effort indifference points, choice data were fitted using the Palemedes toolbox ([www.palamedestoolbox.org](http://www.palamedestoolbox.org/)), a set of routines implemented in Matlab for analysing psychophysical data . These routines were used to fit a logistic function to choice data, characterised by 4 parameters (α, β, γ, λ).


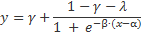


We used free parameters for threshold (α) and slope (β). Fixed parameters were used for the guess and lapse rates, based on the veridical distribution of choice responses for each participant, with γ corresponding to the minimum, and λ being 1 minus the maximum. Thus, the logistic functions fitted for each participant correspond to their actual responses.

**S2. Supplementary Analyses on Patient Data**

**S2.1. Comparison of Indifference Point Slopes**

In the principal analyses described in the main text, the interaction between Drug (ON, OFF) and Stake (1-6) was not statistically significant (*F*(5,125) = 1.26). Graphically, however, there appears to be a potential interaction between the two variables. As suggested by an anonymous reviewer, a more sensitive test for an interaction might be to determine the slope of indifference point curves for individual subjects by regressing them against a non-linear function, and to then compare these slopes in the OFF and ON sessions with a paired *t*-test. Doing so revealed that the slopes between the OFF and ON sessions were not significantly different (*t*(25) = 1.39). This is in keeping with the absent statistical interaction between Drug and Stake, and suggests that the difference between drug sessions is best described as an upward shift of indifference point curves from OFF to ON.

**S2.2. Failure Rates**

As described in the Method section of the manuscript, participants were rewarded only on trials in which they successfully achieved the target effort level. To determine if failure rates differed as a function of Group or Effort, we compared failure rates in patients with a two-way ANOVA on the factors of Drug (ON, OFF) and Effort level (1-6). This analysis revealed a main effect of Effort, such that failure rates increased as a function of effort level (Effort level 1, 1.4 ± 1.1%; Effort level 2, 0.6 ± 0.4%; Effort level 3, 7.2 ± 4.3%; Effort level 4, 32.6 ± 6.1 %; Effort level 5, 60.2 ± 8.6%; Effort level 6, 98.0 ± 2.0%, *p* < .001). Critically, however, neither the main effect of Drug nor its interaction with Effort was significant (both *p* > .90), demonstrating that failure rates did not differ across the ON and OFF sessions.

We also asked whether failure rates may have been correlated with motor severity as measured on the motor section (Part III) of the UPDRS. We therefore performed a correlation analysis between failure rates and scores on the motor section of the UPDRS in each of the ON and OFF sessions. However, there was no correlation between failure rates and motor scores in either of the two sessions (ON, *r* = 0.07, *p* = .74; OFF, *r* = -0.07, *p* = .75), nor was there a correlation between changes in failure rates (ON > OFF) and improvements in motor scores (ON > OFF) across the two drug sessions (*r* = 0.12, *p* = .56).

**S2.3. Effect of Motor Improvement on Effort Indifference Points**

It is conceivable that the amount of effort patients were willing to exert was correlated with the severity of their motor impairment. To address this question, we examined whether effort indifference points were correlated with the motor section (Part III) of the UPDRS, for each of the ON and OFF sessions separately. Importantly, there was no correlation between effort indifference points and the severity of motor symptoms in either session (ON, *r* = -0.14, *p* = .50; OFF, *r* = 0.03, *p* = .89).

On a related point, could the increase in effort indifference points from the OFF to ON sessions be accounted for by a corresponding reduction in the severity of motor symptoms? This is an important consideration, given that a recent study in PD found that patients performed a higher number of keyboard presses for reward while ON medication relative to OFF, but that this change was related to improvements in motor symptoms . To address this issue, we performed a correlation analysis examining changes in effort indifference points from the OFF to ON sessions against changes in the motor section (Part III) of the UPDRS across those two sessions. Importantly, however, this correlation was not significant (*r* = 0.22, *p* = .28).

In summary, the severity of motor symptoms cannot account for the effort indifference points in either the ON or OFF sessions, nor could improvement in patient symptoms account for increases in indifference points from the OFF to ON sessions.

**S2.4. Patient Force Output Data**

The following analyses were conducted in order to verify that the shifts in indifference points reported in the main text could not be attributable simply to changes in force output.

*S2.4.1. Initial MVCs*

To recapitulate, the analyses reported in the main text showed that the MVC at the beginning of each session did not differ between patients ON and OFF medication, nor did they differ between the patient and control groups overall. In addition, the time-to-peak contraction did not differ between the ON and OFF sessions (OFF 2.56 ± 0.1s vs ON 2.53 ± 0.1s, *t*(25) = 1.04, *n.s.*). This suggested that differences in indifference points ON and OFF medication, and between patients and controls, could not simply be due to differences in motor strength at the beginning of each session.

In addition, we tested for any correlations between changes in patients’ MVCs and shifts in their indifference points. For each patient, we calculated the difference in MVC between the ON and OFF sessions. In addition, we calculated differences in their mean effort indifference point ON vs OFF medication. If increases in MVC accounted for increases in patients’ effort indifference points, we would expect a correlation between these two variables. However, no such correlation was found (Pearson’s correlation coefficient -0.004, *p* = .984).

*S2.4.2. Time-on-task Analyses*

In order to verify that there were no changes in force output **during** the experiment, we compared motor output between the first and second halves of each session (90 trials per half). Importantly, dopamine did not differentially affect patients’ maximal grip force across the first and second halves of each session (Drug, *F*(1, 25) = 2.36; Session Half, *F*(1, 25) = 0.19; Drug × Session Half, *F*(1, 25) = 2.03). Similarly, patients’ time-to-peak contraction did not differ over the course of the experiment as a function of drug (Drug, *F*(1, 25) = 1.24; Session Half, *F*(1, 25) = 0.66; Drug × Session Half, *F*(1, 25) = 1.55).

In summary, these analyses show that there were no significant differences in patients’ motor output at the beginning of each experimental session, nor during the sessions themselves. This therefore excludes the possibility that any shifts of effort indifference points were due to changes in force output.

**S2.5. Effect of Medication Class**

Because our study was aimed at examining the effect of dopaminergic medication in general on decision-making, patients in our study were on different therapeutic regimens (levodopa-containing compounds alone (*n* = 10), dopamine agonists alone (*n* = 5), or combinations of both (*n* = 11)). An interesting question is whether performance on our task differed according to medication subgroup. An ANOVA comparing the between-subjects effect of Medication Subgroup (levodopa only, dopamine agonists only, both) on the within-subjects factors of Drug Session (ON, OFF) and Stake Level (1-6) showed that Medication Subgroup was not involved in any significant main effects or interactions (all *F* < 1.21). Obviously, however, this null result should be interpreted with caution given the small and uneven sample sizes in each subgroup, and it would be useful for future studies to pursue whether drug class has a differential effect on motivation.

**S2.6. Effect of Total Levodopa Equivalence Dose**

In addition, we also asked whether dopamine had a dose-dependent effect on effort indifference points. However, the correlations between mean effort indifference points and levodopa equivalent dose was not significant, even after performing a partial correlation controlling for disease duration (*r* = -0.22, *p* = .29).

**S2.7. Effect of Apathy or Depressive Ratings**

One might predict that patients who were towards the more ‘apathetic’ or ‘depressed’ range on the LARS or DASS respectively may have had lower effort indifference points due to their subjectively lower motivation. We therefore performed correlation analyses between participants’ mean effort indifference points and their scores on the LARS and the Depression subscale of the DASS. However, all Spearman correlation coefficients were not significant (all *p* > .05). One potential reason for this is that our participants all scored within the normal range on these measures, and therefore did not demonstrate the variability that might be required to reveal such correlations.

**Supplementary Table 1.**

Summary of UPDRS scores for patients with PD (means ± SD).

| UPDRS | Patient Scores |
| --- | --- |
| Part I | 7.0 (± 4.4) |
| Part II | 11.2 (± 4.7) |
| Part III (ON) | 21.6 (± 11.7) |
| Part III (OFF) | 31.9 (± 13.6) |
| Part IV | 2.08 (± 3.5) |
| Hoehn & Yahr Stage | 1.85 (± 0.54) |

**References**
